# Car and Hotel Disinfection

**Published:** 1902-09

**Authors:** 


### CAR AND HOTEL DISINFECTION.

**An Ideal Germicide-Gas Generator.**

We have been shown in operation a small furnace for generating a combination of the most deadly gases known to sanitary science, for the thorough sterilizing of infected cars or apartments. It is the invention of Dr. L. W. Cock, of San Marcos, Texas, who has applied for patents on the process and on the device. It consists of a sheet iron cylinder about the size of, and not unlike in appearance, the little coal oil stoves used in bath rooms, etc., in which is a furnace for burning charcoal, sulphur, boric acid and other substances. These are ignited by a methyl alcohol lamp, the flame of which is projected against the hot metal of the grate, thus generating dry formaldehyde gas in the form and manner which is known to be more penetrating than when generated from the forty per cent. solution (formalin). That form is a mere surface disinfectant. The chemistry of the gases set free by Dr. Cock's apparatus is very complicated, and the combination modifies the gas of the sulphur in that it is robbed of its corroding and bleaching properties, the objections heretofore attending the use of sulphur dioxide for general disinfecting purposes. In the test we witnessed, silver, nickel plate, brass, gilt picture frames, etc.; and scarlet velvet, blue velvet and other such fabrics as are used in upholstering cars and furniture, were subjected to the action of the gases all night, and were not injured; nor was the wall paper of the room. The killing property of the gases cannot be questioned: it will kill anything. It is a combination of formaldehyde, sulphur dioxide and carbon dioxide, and other gases. Such apparatus should come into immediate and general use, not only in sleeping cars and day coaches, hotels and boarding houses, hospitals, jails, etc., but in private residences. It will not only thoroughly disinfect a room or car and every thing in it, but will kill flies, ants, chinches, moths, roaches, weevils and all creeping things, and without injury to the fabrics or furniture. It is portable, compact, inexpensive and very thorough in its action. Dr. Cock informs me that it has the official endorsement of the State Health Offijcer, who had witnessed the test referred to, and will be used in the quarantine service. The I. & G. N. Railroad has adopted it also, and will begin its use on their entire system as soon as the generators which are now being made in St. Louis are received by them. They were orderd by advice of their Chief Surgeon in Texas, Dr. W. G. Jameson, Palestine.

It will be remembered that one of the big railroad companies in Texas had to pay recently $15,000 damages for a case of smallpox alleged to have been acquired by the plaintiff by sleeping in one of their sleepers. Prevention is certainly better than cure, as well as less expensive.

